# The DIVER Microscope for Imaging in Scattering Media

**DOI:** 10.3390/mps2020053

**Published:** 2019-06-21

**Authors:** Alexander Dvornikov, Leonel Malacrida, Enrico Gratton

**Affiliations:** 1 Laboratory for Fluorescence Dynamics, Department of Biomedical Engineering, University of California Irvine, Irvine, CA 92697, USA; advornik@uci.edu (A.D.); lmalacri@uci.edu (L.M.); 2 Departamento de Fisiopatología, Hospital de Clínicas, Facultad de Medicina, Universidad de la República-Uruguay, Montevideo 11400, Uruguay

**Keywords:** non-linear microscopy, hyperspectral imaging, FLIM, scattering media, fluorescence

## Abstract

We describe an advanced DIVER (Deep Imaging Via Emission Recovery) detection system for two-photon fluorescence microscopy that allows imaging in multiple scattering media, including biological tissues, up to a depth of a few mm with micron resolution. This detection system is more sensitive to low level light signals than conventional epi-detection used in two-photon fluorescence microscopes. The DIVER detector efficiently collects scattered emission photons from a wide area of turbid samples at almost any entrance angle in a 2π spherical angle. Using an epi-detection scheme only photons coming from a relatively small area of a sample and at narrow acceptance angle can be detected. The transmission geometry of the DIVER imaging system makes it exceptionally suitable for Second and Third Harmonic Generation (SHG, THG) signal detection. It also has in-depth fluorescence lifetime imaging (FLIM) capability. Using special optical filters with sin-cos spectral response, hyperspectral analysis of images acquired in-depth in scattering media can be performed. The system was successfully employed in imaging of various biological tissues. The DIVER detector can be plugged into a standard microscope stage and used as an external detector with upright commercial two-photon microscopes.

## 1. Introduction

The ability to visualize features in deep layers of biological tissue with high resolution is a very sought-after feature of imaging systems employed in medical diagnostics, as well as in clinical and biological applications. Deep-tissue imaging has many applications, all based on the necessity of exploring cells and molecules in the intact organism. As a general goal, it may serve as a kind of “optical anatomo-pathology” tool that removes the requirement of biopsies and tissue fixation and allows access to structures and functions in the native physiological environment. Both geometrical and optical properties of a sample and characteristics of the microscope system affect the achievable imaging depth. While transparent specimens can be easily imaged with a traditional microscope, biological tissue is an intrinsically turbid medium, which produces strong multiple scattering, and absorption and which exhibits inhomogeneity of the refractive index. These features make traditional light and fluorescence microscopy inefficient at depths more than 100–200 μm from the surface of a sample [1], even with the aid of staining and the addition of fluorescent markers to improve the contrast.

In order to access deeper layers of turbid samples, high power short pulsed lasers emitting in near infrared (NIR) are required. This approach allows deeper light penetration into media, because of the reduced absorption and scattering at longer wavelengths. The advent of multi-photon microscopy has improved the achievable imaging depth, but the current microscope technology is still limited to an imaging depth of about 1 mm [2,3,4,5].

We built a two-photon fluorescence microscope, the DIVER (Deep Imaging Via Emission Recovery) that is capable of imaging in turbid media simulating brain optical properties up to the depth of a few mm. Such an increased penetration depth is the result of a novel photon harvesting system which collects emission photons from a wide area of the sample in contrast to the conventional detection scheme that collects photons only from a relatively small area, defined by the objective Numerical Aperture (NA), and therefore the loss of most emission photons. In our microscope light losses are also reduced by means of refractive index matching throughout the optical path. The system has proven effective on imaging of biological and artificial samples, such as murine colon and small intestine, vasculature in the skin, subcutaneous xenograft tumors in mice and various tissue phantoms [6,7,8,9].

Due to its transmission geometry, the DIVER imaging system is also particularly suitable for Second and Third Harmonic Generation (SHG, THG) signal detection. SHG is a coherent process that produces photons at exactly half the wavelength of the excitation light. Second harmonic generation is a useful optical tool for biological and medical imaging and diagnostics [10]. On the other hand, THG is generated at the interphase of a sharp change in the index of refraction [11]. The physical principle for both processes does not involve absorption to generate contrast, like in the case of multi-photon fluorescence microscopy, but arises from the polarization of specific endogenous structures lacking center of symmetry (SHG) or mismatch in the refraction index (THG), thus not requiring staining of the sample. SHG and TGH also work with NIR lasers frequencies, which allows imaging at an increased depth in biological tissue with high resolution. Unlike fluorescence, which produces an isotropic emission, SHG and THG mainly propagate in the direction of the excitation beam [12,13]. Due to its intrinsic forward-directed nature, SHG and THG detection is better accomplished in an imaging system working in transmission geometry. 

SHG is a very powerful imaging technique, both ex vivo and in vivo, and, for instance, has been successfully used in imaging of diseases of connective tissue [14]. Some types of collagen, the most abundant component of the extra-cellular matrix (ECM), generate a very strong second harmonic signal. Many diseases, such as ovarian [15] and breast cancer [16] are characterized by a remodeling of the ECM. Additionally, myosin, present in large amounts in biological samples, gives a strong SHG signal and the imaging of the arrangement of the fibrils can give insight into some muscle diseases [17]. 

We were able to obtain SHG images of biological and non-biological specimens in depth using very low excitation light power, thus causing less photo-damage, when compared to traditional microscope geometry [18,19].

The DIVER system is also equipped with a FLIMBox card that allows in depth fluorescence lifetime imaging (FLIM). FLIM is a powerful imaging technique that maps the spatial distribution of the unique lifetimes of intrinsic and extrinsic fluorophores, such as NADH, FAD, and fluorescent proteins [20,21]. FLIM has many applications to explore various biological processes, such as protein-protein interactions and others [22,23]. Traditionally, FLIM analysis was performed in the time domain and has been computationally demanding [24]. The phasor-approach, introduced by Jameson et al. [25] and later on extended to FLIM data analysis in the frequency domain by Digman et al. [26], has greatly simplified and enhanced data processing.

The phasor-approach was also used for data analysis in hyperspectral imaging. The spectral phasor approach introduced by Fereidouni et al. [27] shows major advantages over classical multicomponent deconvolution to resolve hyperspectral images. In the spectral phasor approach the spectra are transformed to the real and imaginary components of the Fourier transformation, then the spectral shift and broadening of the full width at the half maximum (FWHM) are related to the phase increase or modulation decrease, respectively [28,29]. Unlike deconvolution, the spectral phasor approach does not require the knowledge of a spectral basis set for the analysis, which are instead needed for a classical fitting approach [29,30]. In particular, the application of spectral phasors to quantify dipolar relaxations at the interphase of membranes using LAURDAN fluorescence is important in simplifying the study of membrane dynamics and structures in cells and tissues [30,31]. 

Recently we have shown that using the set of two filters with the sine and cosine spectral response it is possible to resolve spectral components in hyperspectral images with single channel detectors, such as the DIVER, and in strongly scattering systems [32]. This technique is particularly useful for hyperspectral imaging in biological tissues, where emitted photons are coming from all directions and hyperspectral detectors, which require collimated light, are not very efficient.

## 2. Materials and Methods

Tissue phantoms imitating brain optical properties and used in experiments were prepared according to [9,33].

Tissue samples, such as mouse brain, rat lungs, mouse bone, liver, kidney, sciatic nerve, adipose tissue or bacteria were generously provided by various research laboratories and researchers working at Laboratory for Fluorescence Dynamics (LFD) on other projects. 

Cyan and Green plastic filters (Amazon, color filters, cat# B016Q0BA6A) were used as cosine and sine filters in hyperspectral imaging experiments. 

Data acquisition and analysis was performed using the Globals for Images-SimFCS software by G-Soft Inc. (Champaign, IL-USA). 

The concepts of DIVER detector operation can be found in [7,8]. Originally it was designed for the purpose of deep imaging in turbid media, particularly in biological tissues, but later it was successfully used in various projects related to diseases in organs, such as kidney, liver and others, owing its exceptional ability to utilize SHG, THG, FLIM and recently for Hyperspectral imagining [9,19,32,34]. Here we present the recent advances in the DIVER imaging system. 

**Figure 1:**
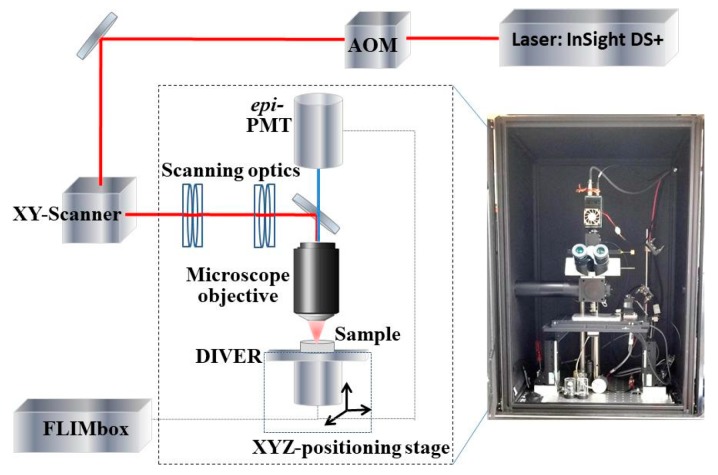
Schematics of the DIVER (Deep Imaging Via Emission Recovery) imaging system.

The custom made DIVER microscope and its key components are schematically shown in Figure 1. It utilizes the Spectra Physics InSight DS+ femtosecond laser for two-photon excitation, tunable in the spectral range of 680–1300 nm. An Acousto-optical Modulator (AOM) by AA Opto-Electronics (Orsay-France), is used to adjust the excitation beam power. XY-Scanner, Cambridge Technology (Bedford, MA, USA) is coupled to scanning optics, constructed using four achromatic doublets (Edmund Optics, Inc, Barrington, NJ, USA) in symmetric arrangement (Plössl-type scan lens), to minimize aberrations and allow wide field of view imaging [35]. This combination of 15 mm clear aperture scanning mirrors and 50 mm diameter lenses allows us to achieve 2–3 fold wider field of view (FOV) compared with commercial microscopes, using the same objective lenses for imaging. 

**Figure 2:**
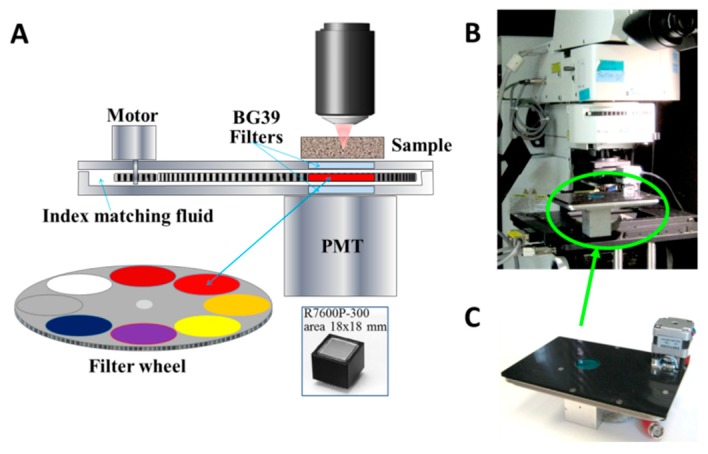
(**A**) The DIVER detector construction. (**B**) Picture of the commercial Olympus BX61 microscope with the DIVER detector. (**C**) Picture of the DIVER detector that can be plugged in the microscope stage.

The DIVER detector, with construction details shown in Figure 2A, is placed in this custom imaging system on the three-axis FTP-2050-XYZLE microscope stage (ASI, OR- USA) equipped with linear encoders for high precision sample positioning. The imaging system is also equipped with an H7422P-40 *epi*-detector (Hamamatsu Photonics K.K., Japan). Both the DIVER and *epi*-detector are connected to a 320 Fast-FLIMbox (ISS, Inc., Champaign-Illinois, USA) to allow FLIM measurements and phasor analysis. The DIVER detector can be directly plugged in microscope stages and used as an external detector in commercial upright microscopes, such as for example Olympus BX 61, as shown in Figure 2B,C.

In the DIVER detector, a photomultiplier tube (PMT) with a wide 18 × 18 mm photocathode area (R7600P-300, Hamamatsu) is used. The PMT is attached to the sealed chamber containing filters/shutter wheel (see Figure 2A). The chamber is filled with propylene glycol to eliminate air gaps in the optical path from the sample to detector. Two 25 mm round BG39 filters (SCHOTT North America Inc., Duryea, PA-USA) are used as windows to seal the chamber and also to block the excitation IR laser light. The filter wheel can accommodate up to eight optical filters of 25 mm diameter (one position is used as a light block) that can be easily exchanged by users. The filter wheel is rotated by the stepper motor to the desired position; the operating software allows automatic sequential image acquisition using different filters and laser powers.

Samples are placed directly on the top window of the DIVER detector. A drop of water is used to fill the air gap between the sample and detector window to reduce reflections; for the same reason a drop of index matching microscope oil is used to fill the gap between the PMT and the bottom window of the chamber. Matching index of refraction in the optical path from a sample surface to the PMT window maximizes photon transmission, which otherwise significantly decreases due to reflections on interfaces. This approach is especially important for efficient collection of scattered emission photons that are propagating at various directions and angles. If there is an air gap between optical elements, photons arriving at critical angle will be lost due to total internal reflection. 

This simple detector construction has proven to be very efficient for collection of scattered emission photons from a wide area of sample surface and practically at any entrance angle in a 2pi solid angle. This feature significantly enhances detector sensitivity when compared with an epi-detection scheme, where most of emitted surface photons are lost, especially when imaging in scattering media. 

## 3. Results

### 3.1. Imaging in Scattering Media: the DIVER Versus Epi-Detector

**Figure 3:**
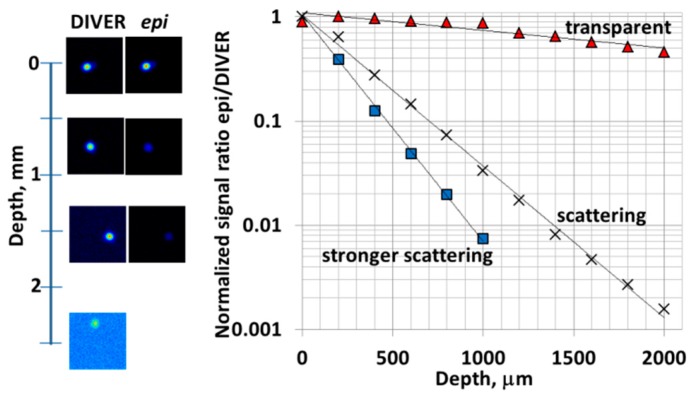
Comparison of the DIVER and the epi-detector efficiency. Images of fluorescent beads in scattering sample (**left**) and dependence of epi- to DIVER detector signals ratio on imaging depth (**right**). Samples measured: transparent (no TiO_2_ particles added); scattering (scattering coefficient μ_s_ = 3.5 mm^−1^); stronger scattering (μ_s_ = 10 mm^−1^).

To compare imaging performance of the DIVER detector and the epi-detector we imaged fluorescent beads embedded in scattering silicone samples, containing Titanium oxide particles for scattering. The scattering properties of these samples were adjusted to match scattering properties of biological tissues, for instance brain tissue (Figure 3 caption).

The images at different depths were acquired at the same time by both detectors and then the ratio of signal intensities was plotted as a function of imaging depth, as shown in Figure 3. At the top surface of the sample the signal intensities were set to be about the same for both detectors. 

With increase of imaging depth in scattering samples the performance of DIVER detector was better than the epi-detector; moreover, at certain depths, images can be acquired only with the DIVER detector. This imaging depth dependence is more pronounced in stronger scattering samples. For transparent samples the DIVER detector also performs slightly better with imaging depth increase, due to the fact that the microscope objective focal point moves toward the DIVER detector. This aspect in turn increases the effective Numerical Aperture of the DIVER detector, while for the epi-detector it remains the same.

**Figure 4:**
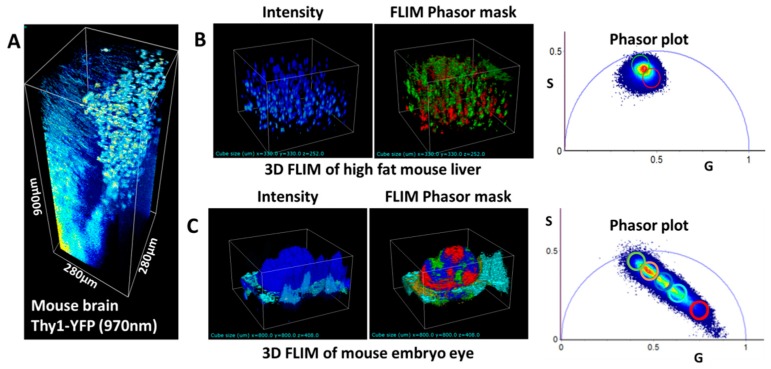
(**A**) 3D reconstitution of a brain section of a transgenic mouse expressing Thy-1-YFP-16 as a marker of neurons. (**B**) 3D intensity and fluorescence lifetime imaging (FLIM) images of autofluorescence from liver tissue of a mouse under high fat diet. The phasor plot allows isolation of different lipids (green and red cursors). (**C**) 3D intensity and FLIM images of a GFP expressing mouse embryo eye. The cursor selections used at the phasor plot enable isolation of different features, such as collagen layers, GFP cells etc., due to lifetime heterogeneities, which cannot be seen in an intensity image.

Figure 4 shows examples of images acquired with the DIVER detector in various tissue samples. The achievable imaging depth depends on tissue optical properties, however, we found that usually the same samples could be imaged 2–3 times deeper using the DIVER system than using the epi-detector or commercial microscopes (like Zeiss LSM-710 or Olympus LSM-BX61). For example, using the DIVER detector we could image the fixed mouse brain tissue from a transgenic mouse expressing Thy-1-YFP-16, which is a motor, sensory and central neuron marker, up to 0.9 mm depth, Figure 4A, while using the epi-detector the imaging depth was limited to about 0.3 mm. 

As was mentioned above, the DIVER detector is coupled with the Fast-FLIMbox which allows acquisition of 3D FLIM images, where various sample features are colored according to their fluorescence lifetimes. Examples of such 3D FLIM images are shown in Figure 4B,C, where features are highlighted by cursors on phasor plots, while intensity images do not reveal these differences. This feature is a quite unique option of the DIVER imaging system. 

**Figure 5:**
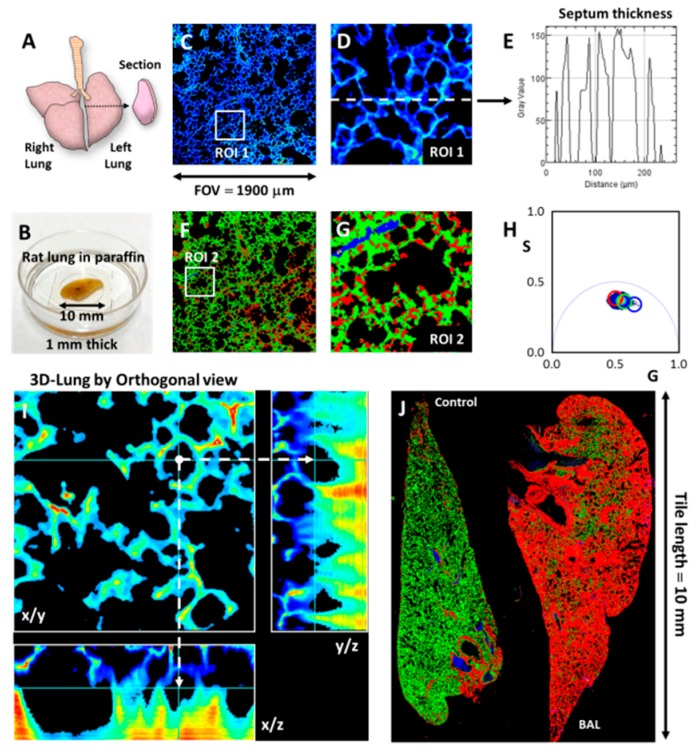
FLIM-Phasor Lung analysis of NADH autofluorescence. (**A**) Illustration of a rat lung and the region from where the section for paraffin inclusion was obtained. (**B**) Picture of the rat lung section included in paraffin and placed in the dish for imaging. (**C**) Large field of view (FOV) FLIM image of lung using Zeiss ECPlan-NEOFLUAR 10x/0.3 objective. The region of interest (ROI 1) is the region shown in section D. (**D**) The dashed line is showing the profile of intensity to quantify the septum thickness. (**E**) Septum thickness plot. (**F**) Pseudocolor image generated by the cursor selection at the phasor plot in H. (**G**) Zoom in of the ROI 2 in (F). (**H**) FLIM-Phasor plot. The cursors select different lifetimes. (**I**) Orthogonal views of the lung 3D image. The arrows indicate an alveolus. (**J**) Pseudocolor images of control and injured rat lungs by broncho-alveolar lavage. The colors indicate differences in the lifetime.

Another example of the potential use of the DIVER microscope and its FLIM capability is application to complex tissue diseases, such as, for instance, lung pathology. Figure 5A–F shows images of a large section (10 × 6 × 1 mm) of rat lung embedded in paraffin, and this sample was also used for traditional microtome cutting. The images were acquired at large ~2 mm FOV. Figure 5D,E shows an enlarged region of interest (ROI 1) from Figure 5C and an intensity profile plot along the dashed line. These images rely only on two-photon induced autofluorescence of NADH and do not require any staining of a sample. The intensity profile plot thus can be potentially used as a structural parameter for anatomical characterization of the septum thickness or alveolar collapse, which are common markers in lung anatomy-pathology procedure, traditionally performed with hematoxylin-eosin staining. In addition, the lifetime heterogeneity can be used to generate contrast and identify modifications in the autofluorescence fingerprint of the lung (Figure 5F–H). The paraffin lung block is perhaps one of the most challenging samples to obtain 3D images, because alveoli are bags of air that make this tissue sample very inhomogeneous and strongly distort images. A 3D z-stack was acquired, and orthogonal views are shown in the Figure 5I. As a result of image distortion, the image resolution decreases with imaging depth, however, the DIVER detector is still able to image in reasonably deep layers. 

Figure 5J illustrates the potential use of autofluorescence images and FLIM-Phasors to fingerprint lung pathology by differences in emission lifetimes for healthy and diseased tissues. In this example we show the difference between a control and a broncho-alveolar lavage injured lung. Using the DIVER microscope ability to image at large FOV we were able easily to acquire images of full lung samples of 10 × 6 mm size.

### 3.2. SHG and THG Imaging

Harmonic generation imaging is a label-free imaging technique, wherein certain molecular structures can generate light of doubled (SHG) or tripled (THG) frequency of excitation light. In biological tissue SHG can be used for imaging features such as collagen fibers, muscles, blood vessels and some other tissues [18,36,37,38]. THG, which is induced by features with sharp changes in refraction index, can be used, for instance, for imaging lipid droplets and cell membranes [39,40]. Both SHG and THG photons are, in most cases, intrinsically forward directed, therefore epi-detection is not efficient for this kind of imaging and requires much higher excitation laser power to induce sufficient photons for detection. Additionally, in conventional multiphoton microscopes utilizing Ti:Sapphire lasers, which are tunable in the ~680–1060 nm range, THG imaging becomes problematic, because of the short THG wavelength (350 nm max) that has to be passed through microscope optics, mainly designed for the visible wavelength range. That is why most THG imaging is performed using special longer wavelength short pulsed lasers.

The DIVER detector has a transmission geometry, where emission photons are induced from one side of a sample and collected from the opposite side. That makes the DIVER microscope very suitable for SHG and THG imaging, since these photons naturally propagate toward the detector. The ability of the DIVER detector to collect photons from the wide area of scattering samples makes it even more preferable for this type of imaging [8,37]. Since the DIVER detector has very few optical elements and its spectral response is defined by the PMT and filters used, it detects photons in the ~320–650 nm range. Thus, with the DIVER detector THG imaging can be easily performed using Ti:Sapphire lasers. 

**Figure 6:**
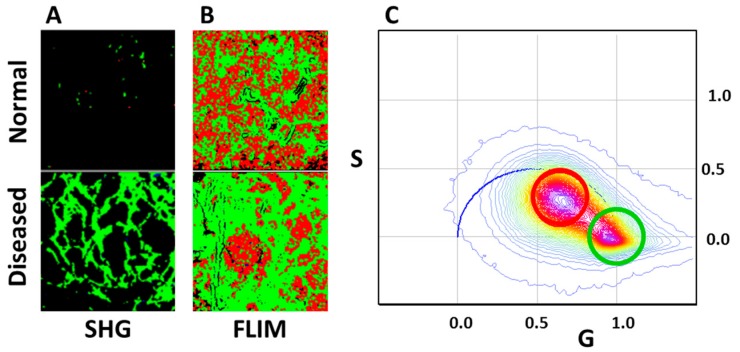
Second Harmonic Generation (SHG) (**A**) and FLIM (**B**) images of normal and diseased kidney samples imaged with the DIVER detector. FLIM images are colored according to cursors in the phasor plot (**C**).

SHG imaging in combination with FLIM using the DIVER detector was successfully applied in kidney fibrosis and diabetes studies [19,34] where progression of the disease could be detected by this method in earlier stages than by standard histology. Figure 6A shows an example of SHG images of collagen fibers in healthy and diseased kidney tissue samples. The normal and diseased tissues can be also recognized by their FLIM autofluorescence signature and SHG on the phasor plot. 

**Figure 7:**
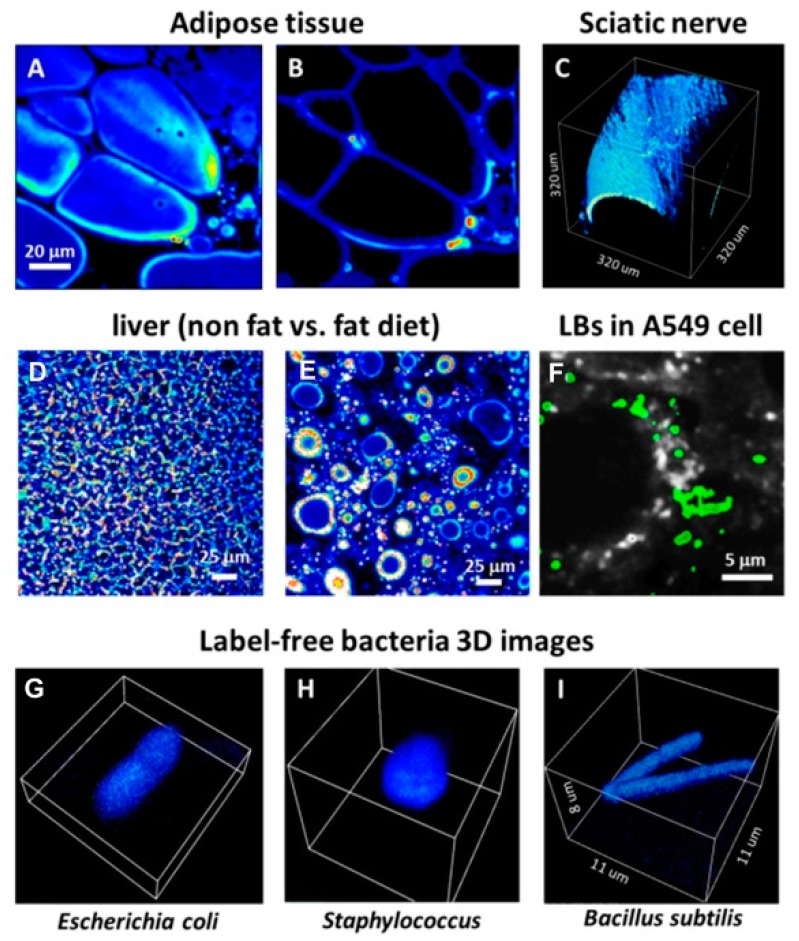
Third Harmonic Generation (THG) images. (**A**) Cell membranes and (**B**) interfaces between cells in adipose tissue. (**C**) 3D image of a mouse sciatic nerve. (**D**) and (**E**) lipids deposition in liver from non-fat or fat diet mouse respectively. (**F**) Overlap of THG signal (green) and NADH autofluorescence from in vivo A549 cells. The green structures refer to lamellar bodies (LBs). (**G**–**I**) 3D-THG images of various bacteria.

Examples of THG imaging are shown in Figure 7, where 1050 nm light was used for imaging and an UG11 filter used to separate 350 nm THG signal.

THG does not involve molecules in excited states and thus allow imaging of features that do not fluoresce, for example, such as lipid deposition in label-free tissues and cells. Figure 7A,B shows THG images of mouse adipose tissue. The cell membranes emit THG photons when they cross a focal point of the scanning IR light. Scanning inside the cells induces THG at edges of the adipocyte, but not in the interior where the refractive index is homogeneous. In other examples of THG imaging we used sciatic nerve and liver from mouse (Figure 7C–E). THG imaging reveals the effect of high fat and normal diets on a mouse liver. Using autofluorescence and THG imaging of A549 cells it was possible to show the presence of lamellar bodies in the interior of in vivo cells (Figure 7F). Lamellar bodies are subcellular organelles associated with the accumulation and secretion of pulmonary surfactant, a substance mainly composed of phospholipids associated with the reduction of pulmonary surface tension [28]. Figure 7G–I shows THG images of label-free bacteria in vivo, where bacteria were in an agarose matrix to avoid movement. 

### 3.3. Hyperspectral Imaging Using Two Filters 

Hyperspectral imaging is a common technique in fluorescence microscopy to obtain the emission spectrum at each pixel of an image. However, methods to obtain spectral resolution based on diffraction gratings or integrated prisms work poorly when the sample is strongly scattering. Spectral phasors were recently introduced as an alternative to spectral demixing, for the determination of FRET and for the measurement of dipolar relaxation of probes in membranes [1,2,3,4,5,6,7,8]. The basic concept in the spectral phasor approach is that the entire spectrum is not needed for some of the classical spectral analysis techniques such as demixing, but rather only a few parameters of the spectral distribution are sufficient for these calculations. Using the properties of the spectral phasors we can resolve spectral components and perform the type of data analyses that are usually performed in hyperspectral imaging.

Recently we have shown [32] that spectral phasor coordinates G and S can be directly calculated from intensity images, passed through optical filters with transmittance spectra “similar” to sine and cosine functions, using the following equations:

I_cos_ = (Σ_λ_F_cos_(λ)I(λ))/Σ_λ_I(λ)

I_sin_ = (Σ_λ_F_sin_(λ)I(λ))/Σ_λ_I(λ)

G = 2(I_cos_ − F_cosMIN_)/(F_cosMAX_ − F_cosMIN_) − 1

S = 2(I_sin_ − F_sinMIN_)/(F_sinMAX_ − F_sinMIN_) − 1,

where F_sin_(λ) and F_cos_(λ) are transmittance of “sine” and “cosine” filters at wavelength λ respectively and I(λ) is the total intensity measured without filters. 

**Figure 8:**
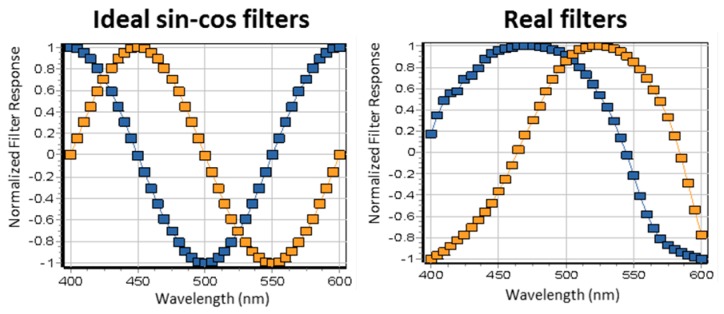
Ideal (**A**) and real (**B**) sin-cos filters response after normalization and shifting to give the range of the cosine and sine function.

We used cyan and green plastic filters as “cosine” and “sine” filters respectively, which were cut and placed in the DIVER detector filter wheel. The empty position in the filter wheel was used for measuring the total intensity images. Filters spectra are shown in Figure 8B. These filters are not ideal; however, they resemble cos-sin functions within the 400–600 nm spectral range and can be corrected for the non-ideal response using correction factors [32]. 

**Figure 9:**
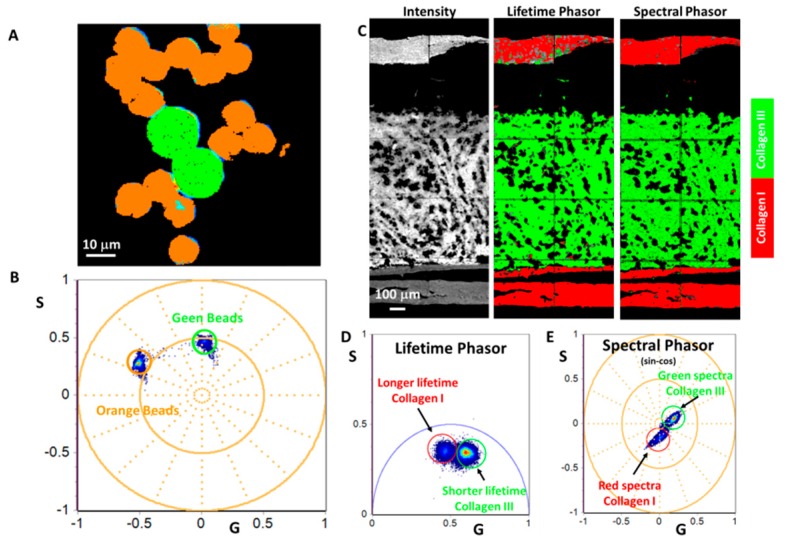
Application of hyperspectral imaging using the sin-cos filters and the spectral phasor transformation. (**A**) 15 μm Yellow-Green and 10 μm Orange fluorescent beads in a highly scattering matrix. (**B**) Spectral phasor plot of beads in (A): using orange and green cursors it is possible to separate different types of beads. (**C**) Images of mouse femur bone. (**D**) FLIM phasor. (**E**) Spectral phasor acquired with sin-cos filters. Both FLIM and spectral phasors allow identifying areas with different kind of collagens (I and III).

Figure 9 shows images and corresponding spectral phasors of fluorescent bead and tissue samples, acquired using cos-sin filters. Orange 10 μm and Yellow-Green 15 μm fluorescent beads are clearly separated in the phasor plot and colored according to cursors in the corresponding image. We have shown earlier [18] that collagen I and collagen III area of the mouse femur bone sample (Figure 9C), can be separated using the FLIM and SHG imaging approach. We were able to demonstrate the same result using sin-cos filters and spectral phasor analysis. 

## 4. Discussion

We have shown that the DIVER detector, which is simple to construct, may significantly improve the imaging depth in turbid samples. The DIVER detector utilizes two main principles: (1) use of wide area detector that allows collecting scattered emission photons from a wide surface area of a turbid sample; (2) the refraction index is matched in the optical path sample-PMT window to minimize signal attenuation due to reflections on optical interfaces. Implementation of these two principles allows harvesting emission photons much more efficiently than with an epi-detector. The DIVER detector becomes more sensitive than the epi-detector to low level signals with an increase in imaging depth and at certain depths scattering samples could be imaged only using the DIVER detector. 

The transmission geometry of the DIVER detector obviously leads to some limitations for its use, because emission photons, induced from one side of a sample, have to travel to other side of a sample to reach the detector. If a sample has strong absorption, these photons will be attenuated and not detected. However, for most biological tissues the absorption coefficient is low and emission photons are mainly attenuated due to scattering, rather than absorption. In this case they still have a chance to reach an opposite surface of a sample and be detected. It was shown that in multiple scattering media attenuation by scattering is linear with the distance, while in clear media the signal decays proportionally to the square of the distance [9,41]. For this reason, at certain depths the scattering may even “boost” the signal. We have shown that in the absence of strong absorption, scattering samples even of few cm thick can be imaged using the DIVER detector. 

On the other hand, the transmission geometry is very useful for SHG and THG imaging, because induced SHG and THG photons are directed toward the detector. This fact and the DIVER detector ability to collect scattered photons from a wide sample area make it orders of magnitude more sensitive for Harmonic Generation Imaging in turbid samples than an epi-detector [8]. Moreover, THG imaging is easily available with the DIVER detector using conventional Ti:Sapphire lasers for excitation, while with epi-detectors it will require special long-wavelength lasers or microscope optics for Ultraviolet (UV) range. 

We also demonstrated that using inexpensive plastic cyan-green filters we can obtain spectral phasors of samples and use them for hyperspectral image analysis. We note that there are no adjustable parameters involved in the procedure and the only information needed is the transmission of filters in the region of sensitivity of the detector. Fluorescence collected after passing through a strongly scattering media gives the same position of the spectral phasor as for transparent material. This hyperspectral imaging method using sin-cos filters is not specific for the DIVER detector and can be used potentially with any microscope and detector, however application to scattering media makes it a very useful addition to the DIVER system. 

Currently we are working on implementation of other, “perfect” sin-cos interference filters for hyperspectral imaging, as well as using adaptive optics technique to correct the point spread function distortion by turbid media and achieve higher imaging depths. 

## 5. Patents 

**Enrico Gratton, Alexander Dvornikov, Viera Crosignani.** Apparatus and Method for Light Emission Detection for In-Depth Imaging of Turbid Media. U.S. Pat. 8692998.
